# Targeted worker removal reveals a lack of flexibility in brood transport specialisation with no compensatory gain in efficiency

**DOI:** 10.1038/s41598-024-55244-w

**Published:** 2024-02-28

**Authors:** Sean McGregor, Fazil E. Uslu, Mahmut Selman Sakar, Laurent Keller

**Affiliations:** 1 Department of Ecology and Evolution, University of Lausanne, Lausanne, Switzerland; 2 Institute of Mechanical Engineering and Institute of Bioengineering, Ecole Polytechnique Fédérale de Lausanne, Lausanne, Switzerland; 3 Social Evolution Unit, Chesières, Switzerland

**Keywords:** Task allocation, Temperature control system, Thermoregulation, Brood care, *Camponotus floridanus*, Automated tracking, Behavioural ecology, Mechanical engineering, Animal physiology

## Abstract

Division of labour is widely thought to increase the task efficiency of eusocial insects. Workers can switch their task to compensate for sudden changes in demand, providing flexible task allocation. In combination with automated tracking technology, we developed a robotic system to precisely control and spatiotemporally manipulate floor temperature over days, which allowed us to predictably drive brood transport behaviour in colonies of the ant *Camponotus floridanus*. Our results indicate that a small number of workers, usually minors belonging to the nurse social group, are highly specialised for brood transport. There was no difference in the speed at which workers transported brood, suggesting that specialisation does not correlate with efficiency. Workers often started to transport the brood only after having identified a better location. There was no evidence that workers shared information about the presence of a better location. Notably, once brood transporters had been removed, none of the remaining workers performed this task, and the brood transport completely stopped. When brood transporters were returned to their colony, brood transport was immediately restored. Taken together, our study reveals that brood transport is an inflexible task, achieved through the synchronous actions of a few privately informed specialist workers.

## Introduction

Division of labour has been suggested as a driving force responsible for asserting both humans^1^ and ants^2^ as ecologically dominant organisms^3^. Division of labour can provide a significant advantage through increased efficiency, allowing tasks to be completed faster with specialists performing distinct roles^4^. The link between specialisation and efficiency is well documented in human societies and evidence supports the emergence of division of labour in early hominins^5,6^, marking a transition from group living with non-specialised task division to the formation of early hunter-gatherer societies^7^.

The link between specialisation and efficiency is less clear amongst eusocial insects^8,9^. Whilst there are unequivocal examples linking specialisation and efficiency in ants, these are only found in highly polymorphic species. For example, *Pheidole dentata* exhibits diphasic allometry where two morphologically distinct worker phenotypes can be identified with the majors performing relatively few specialised behaviours compared to the minors^10^. During antagonistic interactions with foreign ants, the majors leave the nest to attack, while the minors retreat into the nest until the threat is dealt with^11^. Comparison of the fighting ability of major and minor workers revealed that majors had a significantly higher ‘win-rate’ compared to minor workers^10^, demonstrating that majors are specialised and highly efficient at colony defence. This specialist defensive behaviour has also been reported in *Cataglyphis bombycina*^12^, *Orectognathus versicolor*^13^, *Acantomyrmex notabilis* and *A. ferox*^14^. Additionally, species belonging to the *Cephalotes* and *Colobopsis* genera have taken this defensive specialisation a step further and are well known for their specialised phragmotic majors, which feature elaborate head plates (e.g., *Cephalotes texanus*^15^; *Cephalotes rohweri*^16^) or truncated blocky faces (e.g., *C. fraxinola*^17^; *C. nipponica*^18^), which are used to block entrances and gaps in the nest. In most cases, majors are rarely observed to participate in tasks other than nest defence^14,19^ although additional specialist roles for majors have been identified in a small subset of species including seed-milling, food storage^3^, and prey-transport ^20,21^.

There are many examples of eusocial insects exhibiting flexible division of labour, where workers change task in response to demand^22,23^. For example, in honeybees, experimental removal of both nurses and foragers results in task switching among the remaining workers to compensate for the removed individuals^24–30^. While a number of experiments demonstrate similar behavioural flexibility in ants^31–35^, other studies have shown that targeted removal is not compensated for in all cases^36–38^. Of interest would be to study how colonies react when workers that were specialised in a given task are returned to their colony. Such an experiment has not yet been conducted in social insects.

Intranidal brood transport is an important and highly stereotyped behaviour in ants. Workers frequently transport brood within the nest to locations of favorable temperature and humidity to ensure proper growth and development^39–41^. Whilst this behaviour is highly predictable^42–44^, the mechanisms that coordinate brood transport remain unknown. Because this behaviour involves the synchronous actions of multiple workers, it has been suggested that it involves worker recruitment^45^, but empirical data in *Camponotus fellah* suggests that this is not the case^46^.

To reliably generate consistent and predictable patterns of brood transport, we developed a robotic system that allowed us to program the temperature of independent regions of the nest floor over time (the Ant Nest Temperature Controller (ANT°C)). In combination with automated tracking of individuals, brood transport is an ideal behaviour to investigate the interplay between task specialisation, efficiency, and communication. First, we investigated which workers transport brood, whether these workers are specialised, and if specialisation correlated with enhanced efficiency. Next, we performed experiments to determine how workers obtained information relevant to brood transport and whether this information was communicated. Lastly, we tested if workers flexibly re-allocated their tasks and compensated when brood transporters were removed. For the last part, we used the methodology of workforce removal^9,47^, whereby workers transporting brood were removed from the colony.

## Materials and methods

### Study species

We used six colonies of the Florida carpenter ant *Camponotus floridanus*. Incipient colonies were collected at Upper Sugarloaf Key (24.6583°N, 81.5271°W; Florida Keys, USA) and Long Key (24.4859°N, 80.4926°W) during April 2017. Colonies were reared at constant temperature (26 °C) and humidity (65%) with a 12 h:12 h light:dark cycle. Water, 10% sugar water and 10% honey water were provided ad libitum and colonies were fed once a week with flies and an artificial ant diet^48^.

### Experimental setup

We created two subcolonies (i.e., control and treatment) from each colony that consists of 50 larvae, 50 pupae, 100 workers taken within the nest and 100 workers taken from the foraging arena. We did not use eggs as their small size makes their transport by a worker difficult to detect.

The identity, the orientation and the location of each worker was recorded twice a second using the automated ant tracking system described in^49^. Workers were individually identified by gluing a unique matrix code from the ARTag library^50^ to their thorax using a droplet of solvent-free superglue (Pattex Power Easy Gel) following a brief (< 10s) cold-induced immobilisation on a petri dish placed on ice (2 °C). Modifications made by Stroeymeyt et al.^51^ to the lighting system were included to enhance tag detection. As the weight of the smallest worker in our study was 4.03 mg and the weight of each tag was 0.34 mg, the approximate load was at most 8.4% of body weight. Behavioural analyses using the same tag application^49,51,52^ found that tagged ants exhibited behaviour typical of untagged ants. After tagging, we allowed ants to acclimate to the experimental setup for one night, after which we started automated tracking for 13 days.

Within the tracking system, ants had access to a rectangular nest connected via an opaque tunnel to a foraging arena (both boxes were 250 × 125 mm, with fluon-coated walls). To maximise the contrast between the floor and the ARtag ID tags, the foraging arena had a foam floor (Kramer-Krieg SA, Switzerland). The system was maintained at 65% humidity, with the nest in constant darkness and the foraging arena following a light:dark cycle of 12 h:12 h (06:00:18:00 UTC). Temperature in the foraging arena fluctuated between 23 and 25 °C according to the light:dark cycle. Within the foraging arena, ants were provided ad libitum with water, 10% sugar-water and 10% honey-water through cotton-stoppered plastic reservoirs, and artificial ant diet provided on a small plastic tray. Reservoirs were refilled every other day at 17:00 UTC throughout the experiment.

### ANT°C system

**Figure 1:**
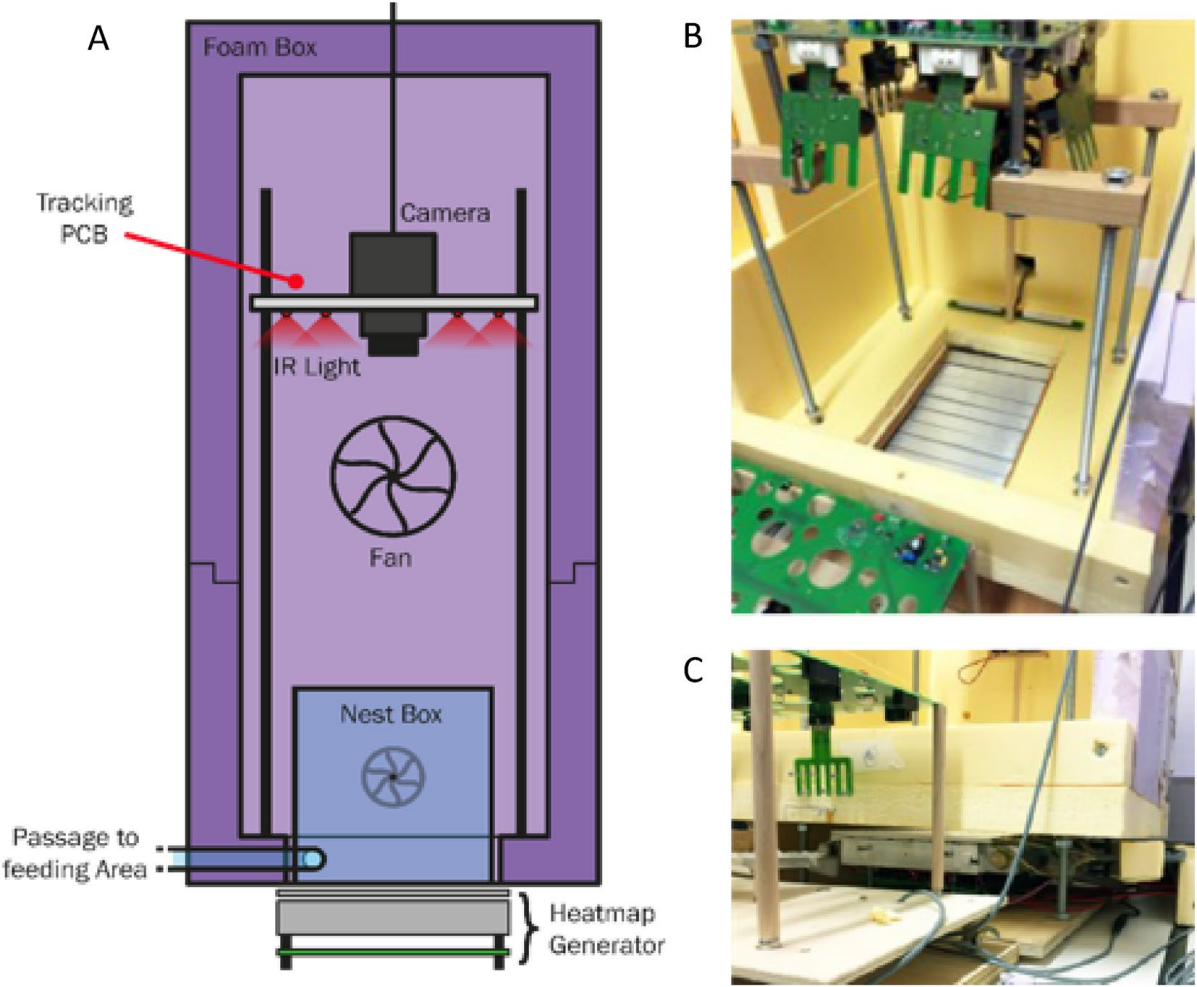
Robotic temperature regulation system. (**A**) Vertical schematic of the nest box of a tracking system with fundamental components comprising tracking camera with a frame capture rate of 0.5 s intervals, pulsed IR lighting to illuminate the otherwise dark interior, fan system to maintain humidity and ambient temperature, and foam insulation to eliminate the influence of external environmental oscillations. (**B**) & (**C**) Images of the ANT°C system implemented in the tracking system.

The surface of the Ant Nest Temperature Controller (ANT°C) made up the floor of the nest (Fig. 1). This robotic system allowed us to generate thermal gradients within the nest corresponding to the colonies’ thermal preferences for the brood.

**Figure 2:**
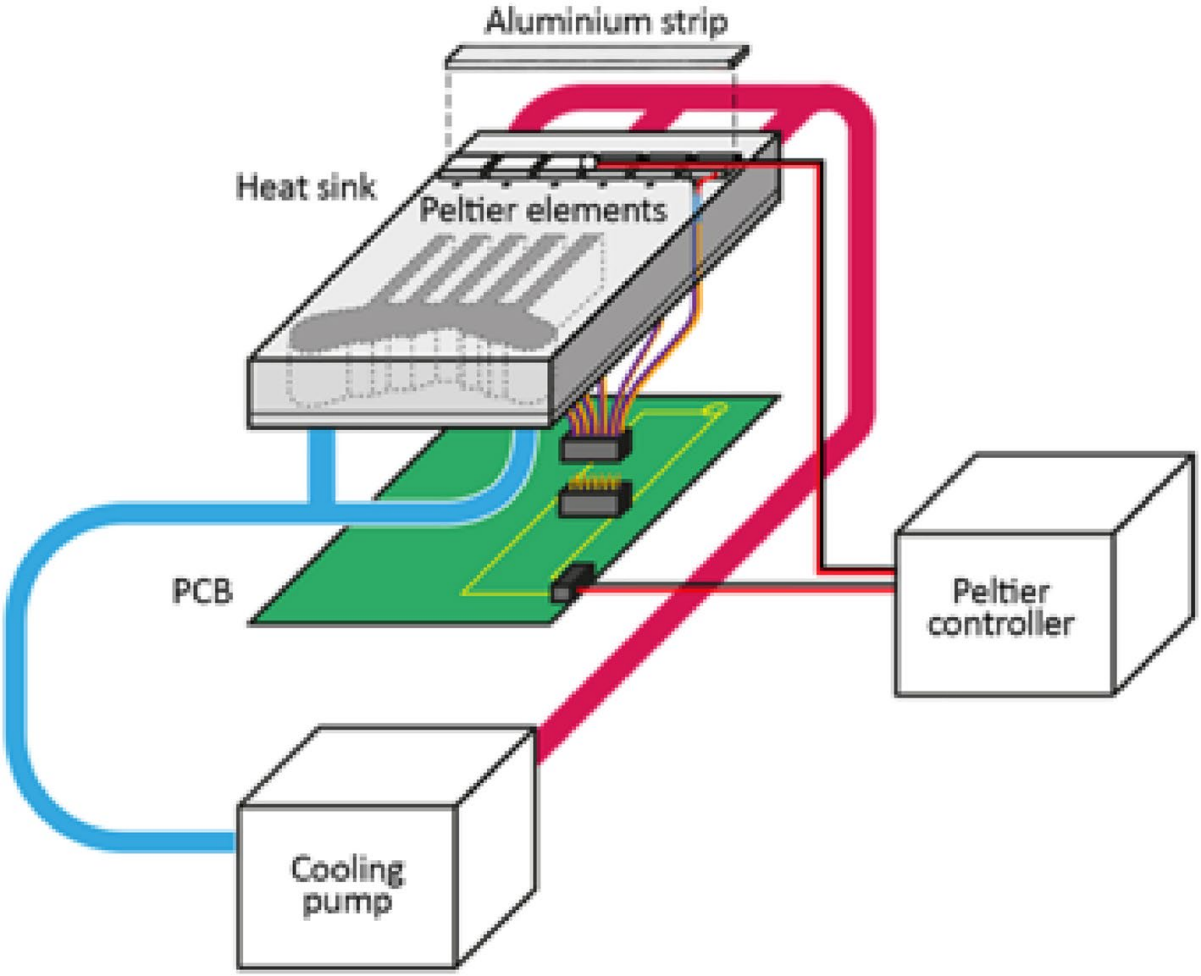
Simplified schematic of the ANT°C system illustrating one of the ten aluminium strips. Each strip rested on top of six-serially connected Peltier elements, which were connected to an integrated printed circuit board below that distributed current from a programmable Peltier controller. The same Peltier controller also connected to an object sensor located above the Peltier elements to provide continuous feedback for automatic temperature adjustment. Beneath the Peltier elements was a heat sink with engraved cooling channels. Cold water was pumped in one end, absorbed excess heat from the sink, and passed back into the cooling pump to be chilled once more.

An in-depth description and explanation of the development and construction of the ANT°C is available in the “Supplementary Information”. In summary, the contact interface of the ANT°C was made from ten aluminium strips mounted on top of 60 Peltier elements (CP60240, CUI Inc.) patterned as a 6 × 10 matrix. Aluminium strips were coated with matte yellow paint to increase the contrast between the strips and the ARtag ID tags, which improved detection. Each set of six Peltier elements was serially connected such that ten identically sized regions of the nest could be independently addressed. Between the surface of the Peltier elements and the aluminium surface strip, a small temperature object sensor (DM-314 PT100 sensor, Farnell) was affixed to feedback the temperature of the strip and ensured accuracy to 0.01 °C. Thermal paste (Arctic) was used to optimise and ensure uniformity of heat transfer from the Peltier elements to the strip above. Below the Peltier elements was an aluminium heat sink with engraved channels for external water cooling supplied by a water-cooling system (Model 157-5251 Arctic circulator, ThermoFisher), which was maintained at 12 °C. Underneath the sink was an integrated printed circuit board (PCB) which directs current from Peltier Controllers (TEC-1122-SV, Meerstetter Engineering) to the Peltier elements (Fig. 2).

### Experimental protocol

**Figure 3:**
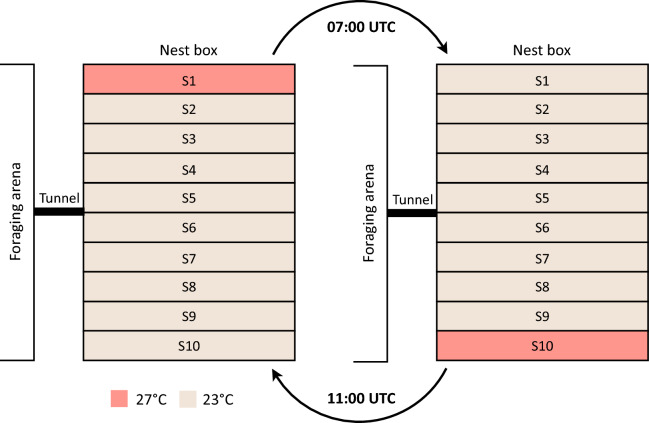
Diagrammatic representation of the nest and foraging arena connected by an opaque tunnel. The nest was divided into ten strips (S1–S10), each of which was controlled at specific temperatures over the course of the experiment: S1 and S10 were 23 °C or 27 °C and S2–S9 remained at 23 °C for the entire experiment.

The initial temperature setup within the nest was 27 °C at strip 1 (S1, Fig. 3), as this is the preferential temperature for brood placement of *C. floridanus*, as determined in our preliminary trials as well as in other studies (Roces, F. pers. comm.). The remaining S2–S10 strips (Fig. 3) were at 23 °C. Each day at 07:00 UTC, the temperature profile of S1 was changed from 27 to 23 °C and S10 was changed from 23 to 27 °C (Fig. 3). Temperature change occurred at a rate of 0.5 °C/s. For 150 min (until 09:30 UTC), the ants were observed via a monitor connected to the tracking system video feed and all brood transport events were recorded manually in real time. If an ant picked up a brood item and transferred it to the 27 °C strip, the identity of the ant and the departure and arrival time were recorded. At 11:00 UTC, the temperature profile was reversed with S1 returning to 27 °C and S10 to 23 °C. Ants were once again observed and brood transports recorded. At 15:00 UTC all ants identified as brood transporters in the treatment subcolony were gently collected using soft entomology forceps and transferred into a husbandry box. An equal number of ants that were not brood transporters were also removed from the control subcolony and transferred into another husbandry box. To minimise light disturbance to the colony, removal was performed with the assistance of a low level red-light headtorch. This procedure was repeated consecutively over ten days. On the tenth day, all ants retained in husbandry boxes up to this point were returned to their respective subcolony. For the next three days, the process described above was repeated except that ants were no longer removed from the subcolonies.

The time taken to transport a brood item was significantly shorter during the second observation session of each day than the first one (*F*(1,5) = 28.546, *p* = 0.003; Supplementary Fig. 2 and Supplementary Table 4). However, as this effect did not vary significantly between days (*F*(9,45) = 1.139, *p* = 0.356) or between subcolonies (*F*(1,5) = 1.010, *p* = 0.361), we therefore averaged the time taken to move brood across the two observation sessions for all analyses.

### Specialisation analysis

To evaluate the effect of worker removal on brood transport, we measured the time taken to move brood to the new 27 °C strip location. As the rate of brood transport over time fitted an approximate sigmoid function (Supplementary Fig. S1), we decided to evaluate the time taken to move half the brood, as this represented a point at which brood transport was close to its maximal rate. If half the brood had not been transported during either of the two observation sessions per day, the time recorded for that session was fixed to 150 min, which corresponded to the duration of one observation session. We performed a Generalised Linear Mixed Model (GLMER; lme4 package^53^) with time taken to move half the brood as the dependent variable, and treatment and day as explanatory variables. Colony identity was considered as a random factor in all analyses. All models were written in the R 4.2.1 programming language^54^. To determine how the re-introduction of workers affected brood transport, we compared the average time taken to move the brood between the three days before (days 8–10) and three days after re-introduction (days 11–13) using a Wilcoxon rank sum test.

Because some workers only transported brood a few times and took a long time to do so, data on mean speed were not normally distributed, which had a strong leverage effect on regression analyses. We therefore categorised workers that transported brood as either infrequent or frequent transporters. Infrequent transporters were workers that transported brood at least once, but during five or fewer observation sessions, which included data points with a non-normal distribution, while frequent transporters transported brood on more than five observation sessions. To assess whether workers transporting brood at a higher frequency were more efficient, we regressed the mean time taken to transport brood against the frequency with which each worker transported brood and the total number of brood transported using Linear Mixed Effect Models (LMER; lme4 package^53^). As the normality assumptions of the LMER model were not met when using data from infrequent transporters, we performed two separate analyses for infrequent and frequent transporters. Comparison of the models found that irrespective of whether we used data from infrequent or frequent transporters, both were highly consistent. This validated the results of the LMER analysis using data from infrequent transporters, despite the non-normal data distribution, as the model conducted with data using frequent transporters gave analogous results and satisfied all requirements.

### Social network analysis

Tracking data collection and pipelines for analysis were automated and therefore not subject to biases. We constructed the social network of our control and treatment subcolonies using physical interactions between ants. These interactions were inferred using the geometric algorithm detailed in Mersch et al.^49^, whereby a trapezoid was calculated for each ant using individual measurements of antennae reach and body length. We adopted a conservative approach whereby any overlap between the trapezoids of two individuals was registered as a physical interaction and the length of the interaction was disregarded. As such, we did not discriminate between potentially distinct forms of interaction. These interactions were then used to construct a weighted network whereby each node represented a worker and each edge a single interaction. Social networks were constructed each day over the 13 day experimental period using tracking data, spanning the 9 h period prior to the first temperature change at 7:00 UTC. As such, the social network was unaffected by any organisational changes that took place as the result of temperature change, and subsequent brood transport, which might have confounded the analysis.

To evaluate the social network structure, we used facetNet (^55^; as implemented in Richardson et al.^52^), which provides several key advantages when analysing dynamic social networks that change over time. First, facetNet uses soft community detection, which partitions nodes (ants) into discrete social groups (communities) based on their interactions, whilst simultaneously allowing a node to belong to more than one community. Second, facetNet outputs a continuous score that quantifies a node’s affiliation to each of the communities detected, and thirdly it permits temporal continuity in the community structure whereby prior node affiliation at time *t*—1 can have a weighted influence on node affiliation at time* t*. In our analyses of community structure, we allowed facetNet to determine the best supported number of communities.

To perform community detection on our social networks we followed the methodology laid out in Richardson et al.^52^. All interactions between individual ants $i,j\in (1,\dots , {\text{n}})$ were recorded in a standard interaction matrix. The observed probability for an interaction ${W}_{i,j}$ was evaluated with the joint distribution ${W}_{i,j}\approx {p}_{i\to k }\cdot {p}_{k}\cdot {p}_{j\to k}$ which evaluates the probability of observing an interaction between ants *i* and* j* within the community $k\in (1,\dots ,{m}_{c})$. As the number of communities ${m}_{c}$ must be designated prior to the implementation of facetNet, we first evaluated modularity allowing for 2, 3, 4 or 5 potential communities and found that modularity was optimised when ${m}_{c}$= 2 (*see also* Richardson et al.^52^). Subsequently, all iterations of facetNet assumed two communities. Therefore, the affiliation (*A*) of a given ant (*i*) to a given community (*k*) was calculated as ${A}_{i\to k}={p}_{i\to k}\cdot {p}_{k}/({\sum }_{k}^{{m}_{c}}{p}_{i \to k}\cdot {p}_{k})$. As we have no *a-priori* knowledge of which community corresponds to nurses or foragers we observed two hours of video captured before the first temperature change each day and classified the first three ants that antennated and/or physically interacted with a brood as nurses and the first three ants that antennated and/or consumed water or a food resource as foragers. Workers were attributed to the community for which they had the highest affiliation score. The community ${k}_{N}$ for which nurse workers showed the highest affiliation score is labelled as the nurse community and the expression ${M}_{i }=1- {A}_{i\to {k}_{N}}$ is the social maturity of an individual $i$. All workers classified as nurses were always attributed to the same community and all foragers to the other. Social maturity values range from 0 to 1 with values close to 0 indicating a worker that is embedded in the nurse community whereas values close to 1 indicating workers embedded in the forager community. As we calculated the social maturity of workers each day, we assigned a temporal weight to the model using the parameter α ($\in (\mathrm{0,1}))$, whereby an individuals *a-priori* affiliation influenced its future affiliation. This process smoothed comparisons of networks over time. As our experiment manipulated social organisation through targeted removal of individuals, it was expected that social organisation would change and so only a weak influence of α was permitted (α = 0.8).

To determine whether social maturity was correlated with the likelihood of brood transport, we used a binomial GLMER to regress social maturity (explanatory variable) against whether they transported brood (dependent variable). Using LMER, we also tested if social maturity (explanatory variable) predicted the number of observation sessions in which workers transported brood and also the total number of brood transported over the experiment (dependent variables).

### Morphological measurements

As *C. floridanus* exhibits diphasic allometry with minor and major workers^56^, we decided to test whether there was any influence of morphology on brood transport behaviour using data from the control subcolonies. For each ant, we selected an image where the ant was at rest. The number of millimetres (mm) from the furthest edge of one eye to the farthest edge of the other eye was used to measure headwidth and the number of millimetres from the tip of the mandibles to the tip of the gaster was used to measure body length. We identified two distinctive allometric relationships between headwidth and body length (Fig. 5). However, as both headwidth and body length of minor and major workers overlapped, we were unable to distinguish between the smallest major workers and larger minor workers. To overcome this problem, we used a quantitative approach with workers < 1.3 mm headwidth labelled as minors and those with headwidth ≥ 1.3 mm as majors. To determine whether minors transported brood more frequently than majors, we first regressed our worker labelling (explanatory variable) against whether they transported brood (dependent variable) using binomial GLMER. Then, we tested whether worker morphology (explanatory variable) predicted the number of observation sessions in which workers transported brood and also the total number of brood transported (dependent variables) using LMER. We also analysed whether headwidth or body length (explanatory variables) predicted brood transport (dependent variable) for both minors and majors separately, using binomial GLMER.

### Private information and social communication

We explored whether workers first acquired information about the presence of a new 27 °C strip before initiating brood transport. Any worker that visited the new 27 °C strip, at least 10 s prior to brood transport, was considered as privately informed. As social interactions drive a number of behaviours in ant colonies^57,58^ we also tested whether social interactions with privately informed workers stimulated brood transport. Any worker that physically interacted with a privately informed worker before brood transport was considered as being socially informed. We regressed (using GLMER) the time elapsed since the start of each observation session at which each worker became privately or socially informed (explanatory variable) against the time elapsed until the first brood transport by the same given worker (dependent variable).

## Results

### Frequent transporters perform the majority of brood transport

**Figure 4:**
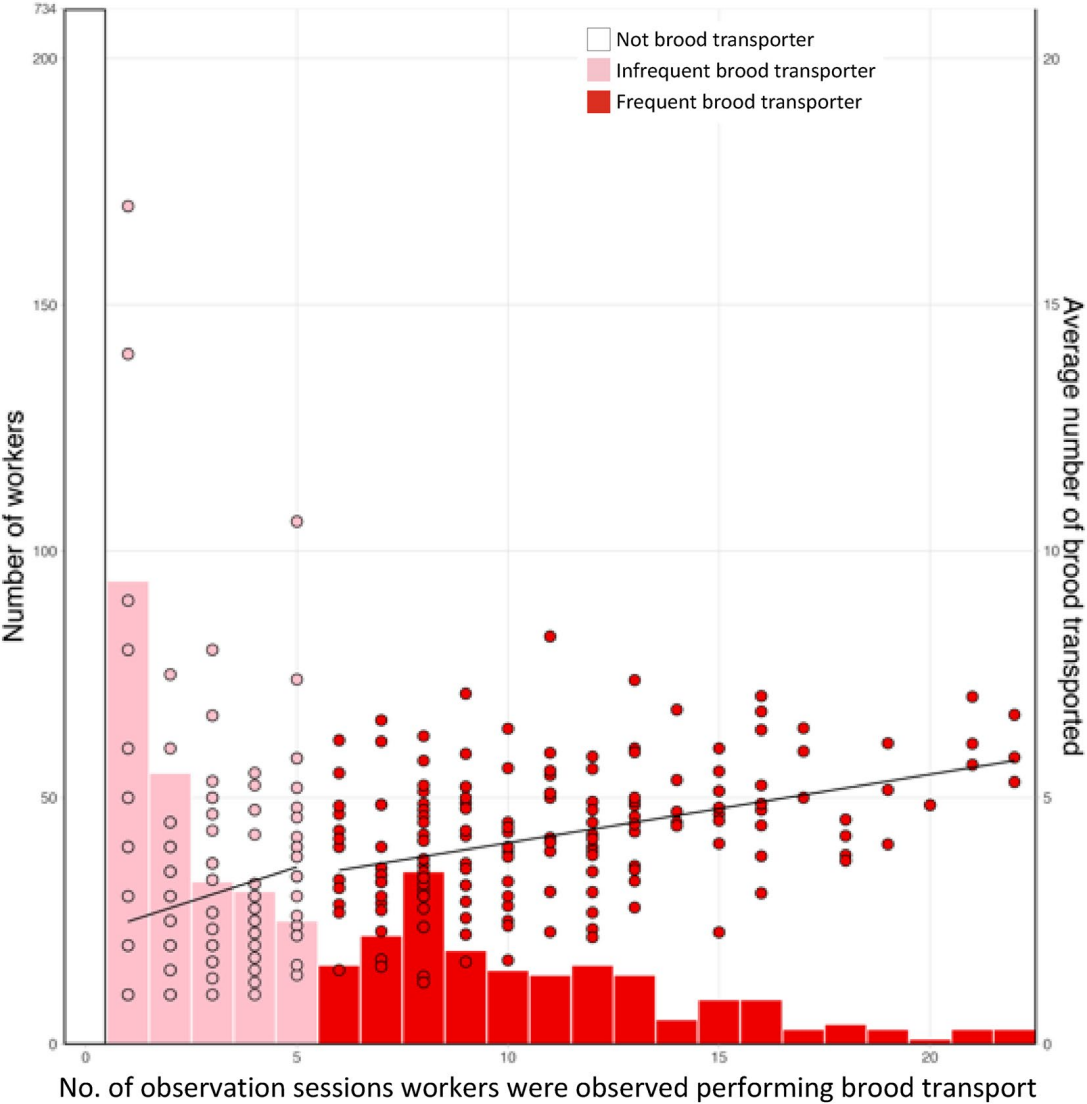
Distribution of workers according to the number of observation sessions in which they transported brood (bars). Correlation between number of observation sessions in which workers transported brood and the per session average number of broods transported (points and lines). Two separate correlations are plotted using data for infrequent (pink) and frequent transporters (red). Data are from control subcolonies.

Relatively few workers transported brood during each observation session (Fig. 4). On day 1, only 6.1% (range 1.0–10.5%) of the workers transported brood during a given observation session (data analysed for each of the 6 pairs of treatment subcolonies where transporters were removed after each session and control subcolonies where an equal number of workers was randomly removed after each session. Initially, they each comprised 200 workers). Using data from control subcolonies only where there was no manipulation of workers performing brood transport, an average of 8.6% of the workers (range: 3.0–15.5%) transported brood on days 2–10. Following the reintroduction of removed workers, this average remained consistent at 8% (range: 2.0–14.5%) on days 11–13. Over the entire 13 days of the experiment, on average 35.7% of the workers (range: 23.5–33.5%) per subcolony transported brood at least once. Of these, on average 19.9% (range: 15–26.5%) were infrequent transporters (≤ five observation sessions) and 15.9% (6–23%) were frequent transporters (> five observation sessions). Overall, an average of 7.4% (range: 3.5–10.5%; Supplementary Table 1) were responsible for half of all the brood transports performed during the entire experiment.

The number of observation sessions during which a worker transported brood was significantly correlated with the average number of brood transported per session by the same given worker. This was true both for infrequent (*t* = 3.59, *p* < 0.0001) and frequent transporters (*t* = 4.06, *p* < 0.0001; Fig. 4 and Supplementary Table 2). The number of observation sessions during which a worker transported brood was also significantly correlated with the total number of brood transported, for both infrequent (*t* = 7.56, *p* < 0.0001 and frequent transporters (*t* = 10.79, *p* < 0.0001; Supplementary Table 2).

### Brood transport is preferentially performed by minor workers

**Figure 5:**
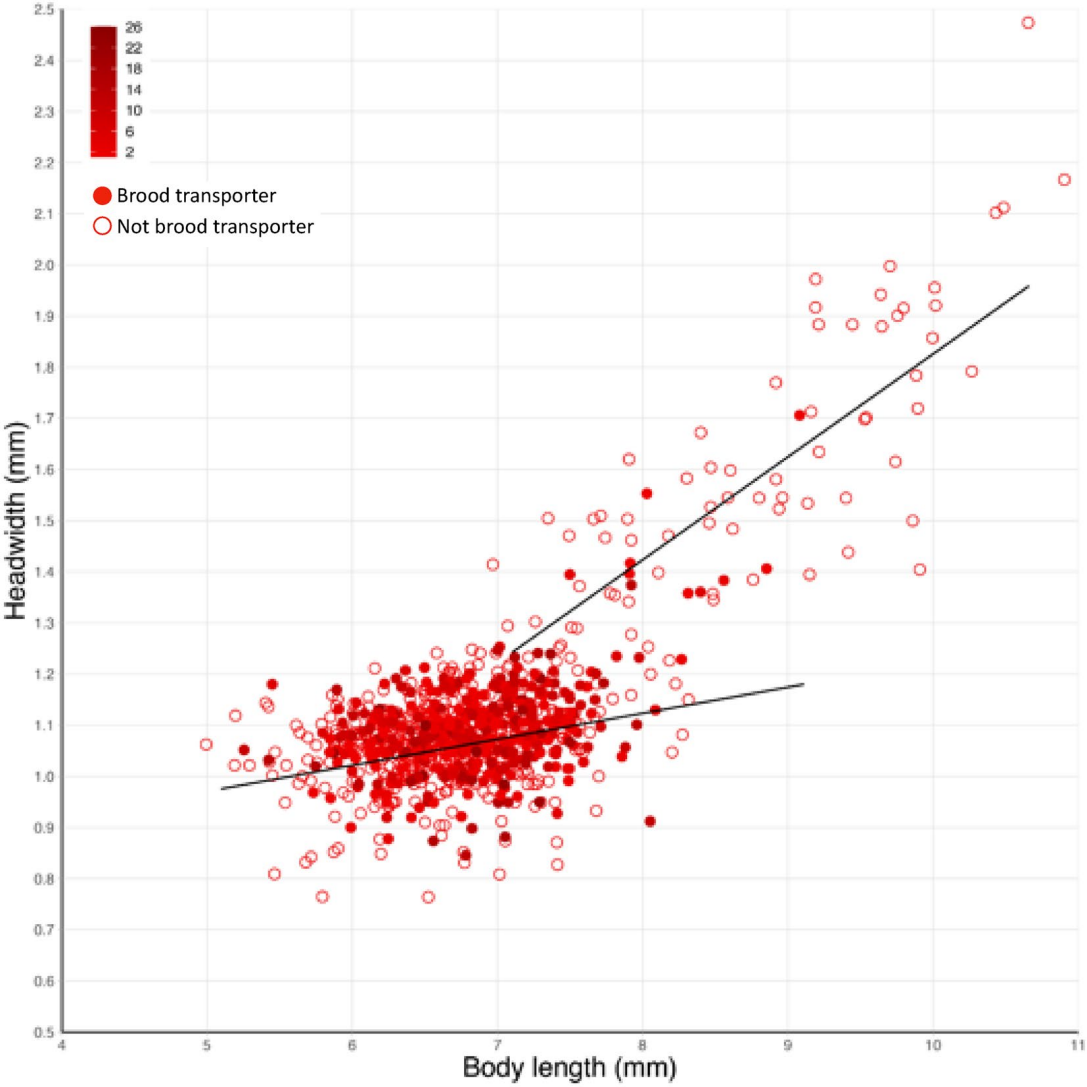
Two distinctive allometric relationships were observed between headwidth (mm) and body length (mm) for minor (lower regression line) and major workers (upper regression line). Filled circles indicate workers which transported brood while open circles indicate workers that did not transport brood. The intensity of the red colour illustrates the number of observation sessions in which workers transported brood. Data are from control subcolonies.

In control subcolonies, throughout the 13 days of the experiment, minor workers were significantly more likely to transport brood than majors (*z* = 7.6, *p* < 0.0001; Fig. 5). Minor workers also transported a significantly higher average number of brood per observation session (control: *t* = 15.33, *p* < 0.0001) and they transported significantly more brood in total (control: *t* = 14.1, *p* < 0.0001) than major workers. Amongst minor workers, there was no significant correlation between headwidth nor body length and the likelihood to transport brood (headwidth: *z* = 1.28, *p* = 0.2; body length: *z* = 0.41, *p* = 0.68). In contrast, amongst majors, headwidth and body length were significantly correlated with the likelihood to transport brood (headwidth: *z* = − 2.29, *p* < 0.05; body length: *z* = − 2.19, *p* < 0.05), such that smaller majors were more likely to transport brood than larger ones.

### Brood transport is preferentially performed by nurses.

Overall, within control subcolonies, the likelihood of brood transport was negatively correlated with social maturity (*z* = − 34.89, *p* < 0.0001). Moreover, the number of observation sessions in which workers transported brood (*t* = − 67.97, *p* < 0.0001) and the total number of broods transported (*t* = − 56.85, *p* < 0.0001) was also negatively correlated with social maturity.

### Brood transporters identify an optimum location for brood before transport

**Figure 6:**
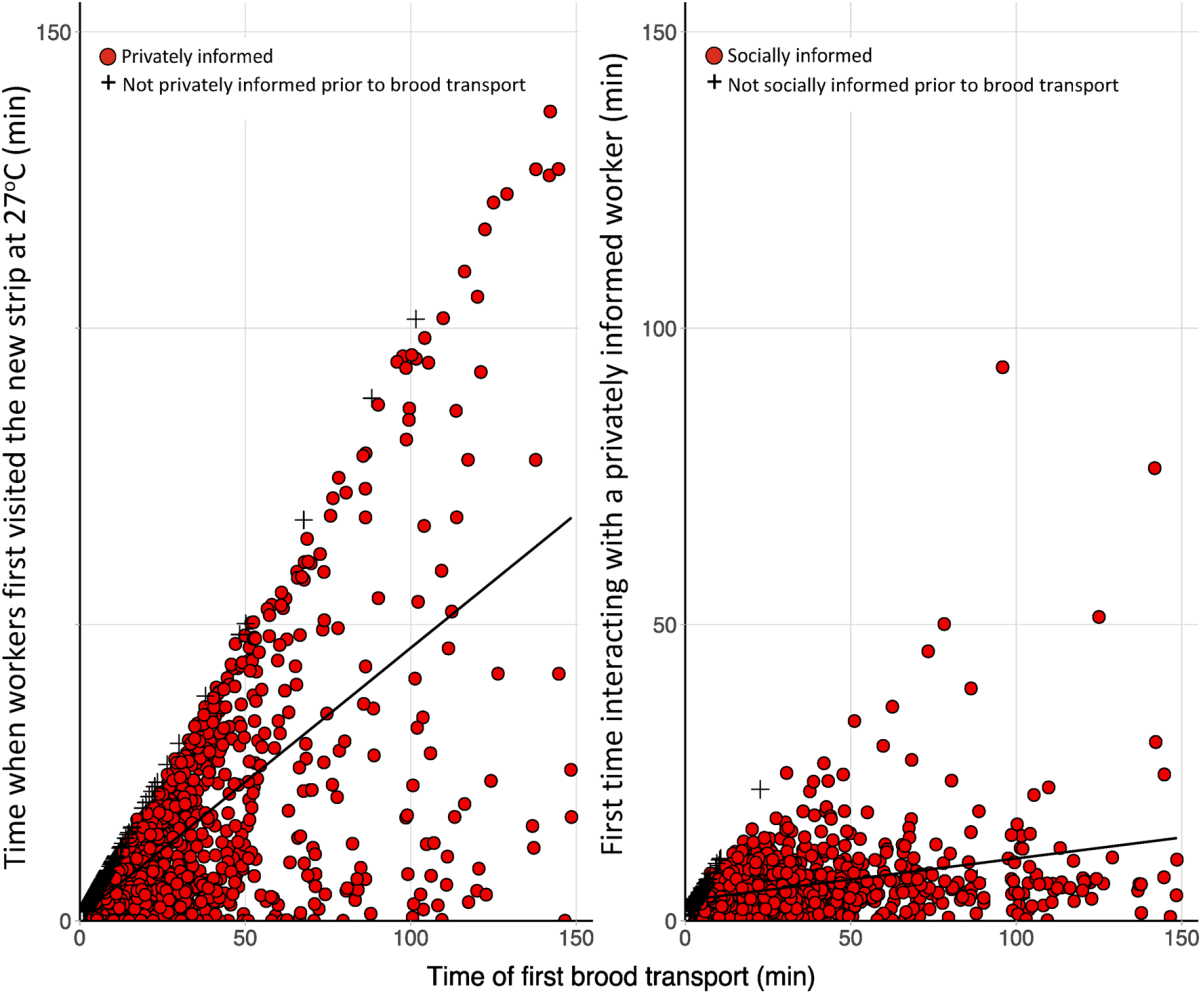
Time elapsed until workers first visited and acquired information about the location of the 27 °C strip (left) and time elapsed until workers first interacted with a privately informed worker (right) against time elapsed until their first brood transport. Filled circles denote transporters which were privately (left) or socially (right) informed prior to brood transport while + symbols indicate workers which were not privately (left) or socially (right) informed prior to brood transport. Data are from control subcolonies.

In control subcolonies the time that elapsed during an observation session before a worker transported its first brood was positively correlated with the time elapsed before that worker was privately informed (i.e., after visiting the new strip at 27 °C; *t* = 43.19, *p* < 0.0001; Fig. 6). In contrast, the time elapsed until a worker transported its first brood was not significantly correlated with the time elapsed until it became socially informed (i.e., interacted with a privately informed worker).

The majority (87%) of the transporters had visited the new strip at 27 °C before transporting brood. Transporters took significantly less time to transport their first brood when they had previously visited the new strip at 27 °C, compared to transporters which had not (*t* = − 3.65, *p* < 0.0005). Furthermore, the less time elapsed during an observation session before a worker had visited the new strip at 27 °C, the more likely it was to subsequently transport brood (*t* = − 21.06, *p* < 0.0001). Whether or not a worker was socially informed, did not affect the speed of the first brood transport (*t* = 0.78, *p* = 0.78).

### No association between specialisation and efficiency in brood transport

**Figure 7:**
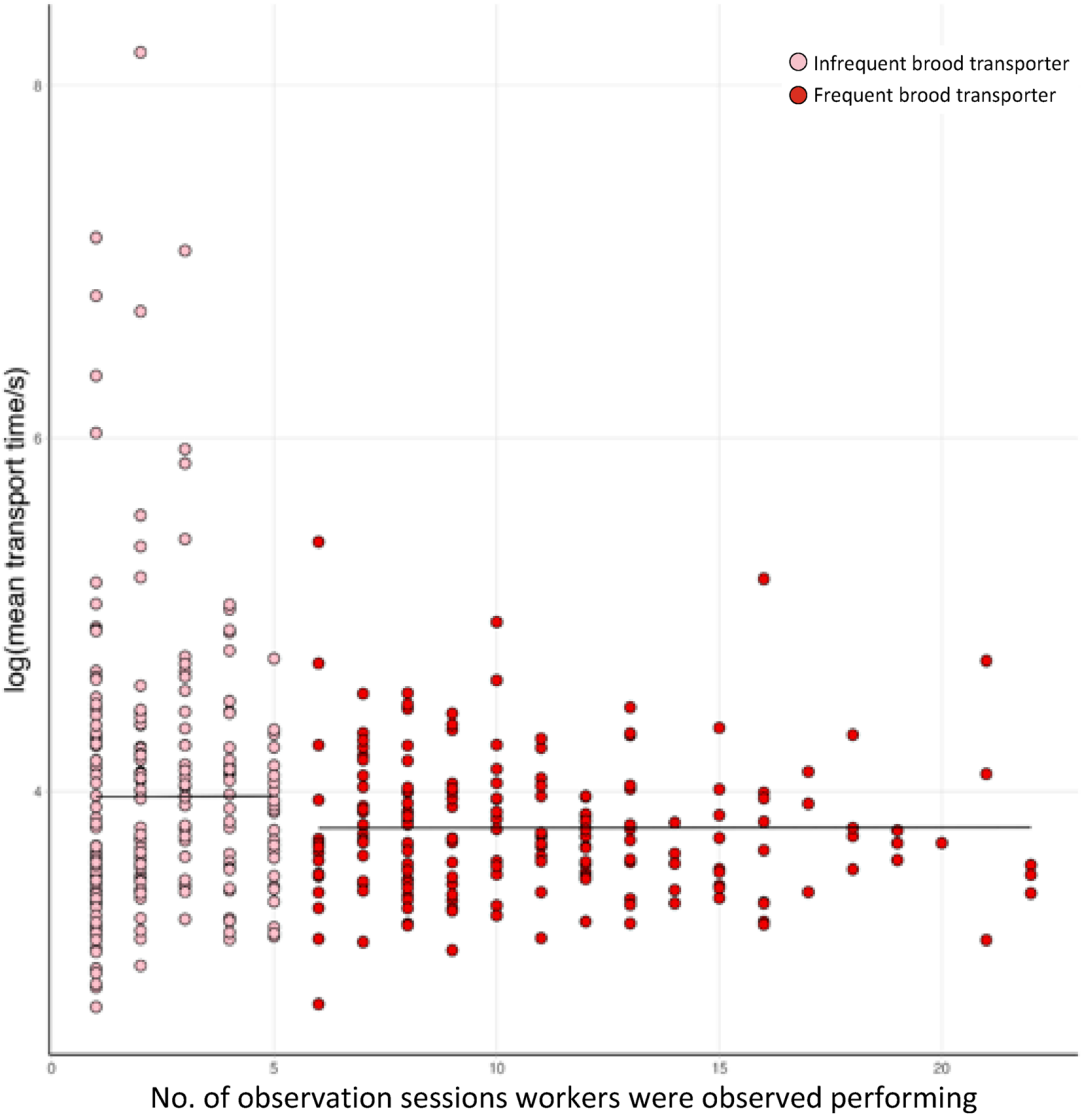
Scatterplots illustrating the mean time (log-transformed) to transport brood as a function of the number of observation sessions in which an ant participated in brood transport. Pink circles are infrequent transporters (≤ 5 observation sessions) and red circles are frequent transporters (> 5 observation sessions). Data are from control subcolonies.

**Figure 8:**
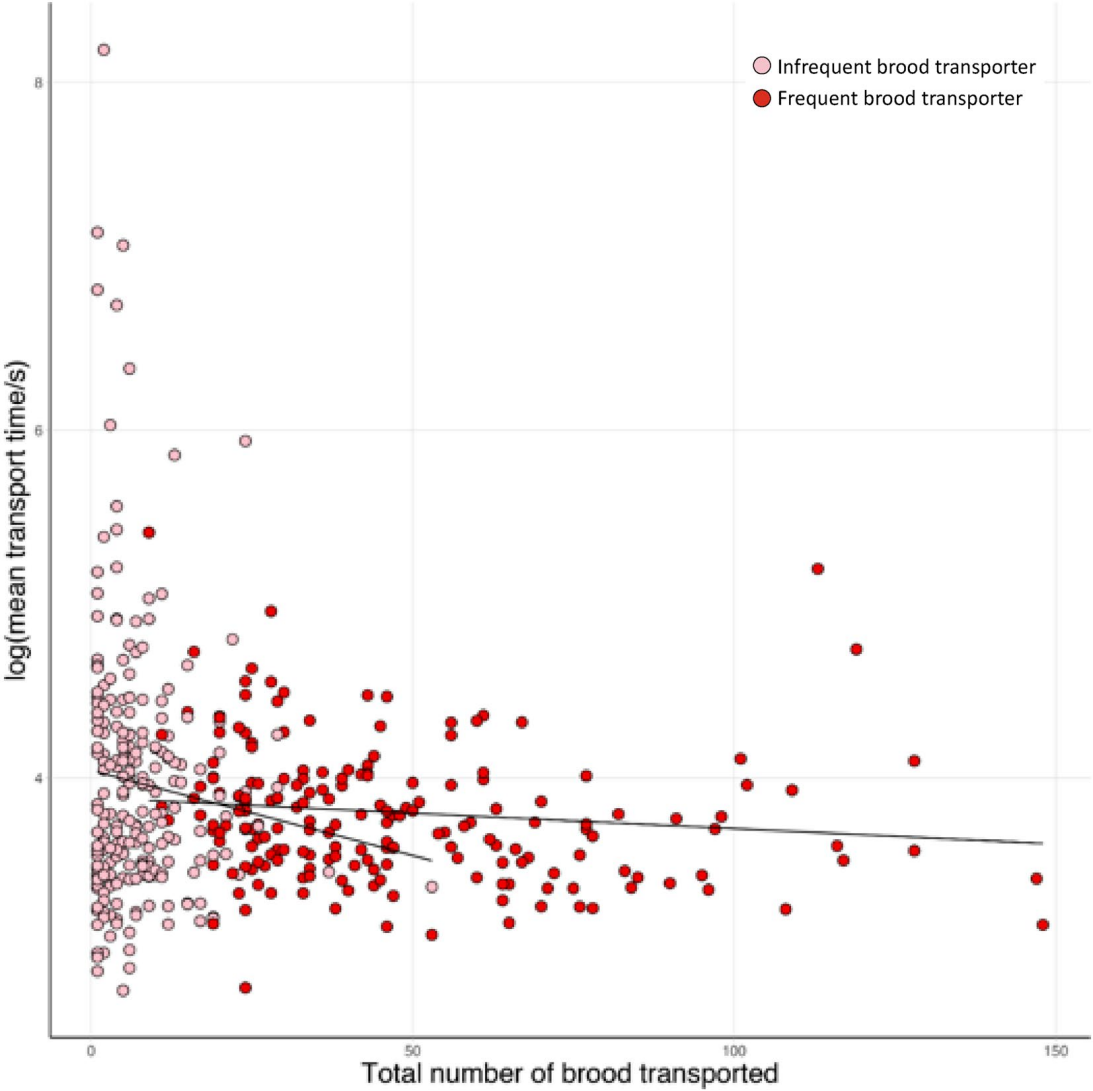
Scatterplots illustrating the mean time for brood transport as a function of the total number of brood transported. Pink circles are infrequent transporters (≤ 5 observation sessions) and red circles are frequent transporters (> 5 observation sessions). Data are from control subcolonies.

Worker efficiency in transporting brood was not associated with their degree of specialisation. In control subcolonies the average time taken to transport a brood item, for both infrequent and frequent transporters, was neither correlated with the number of sessions in which a worker participated to brood transport (Fig. 7 and Supplementary Table 3), nor with the total number of brood transported (Fig. 8 and Supplementary Table 3).

### Targeted worker removal decreases the rate of, and ultimately stops, brood transport

**Figure 9:**
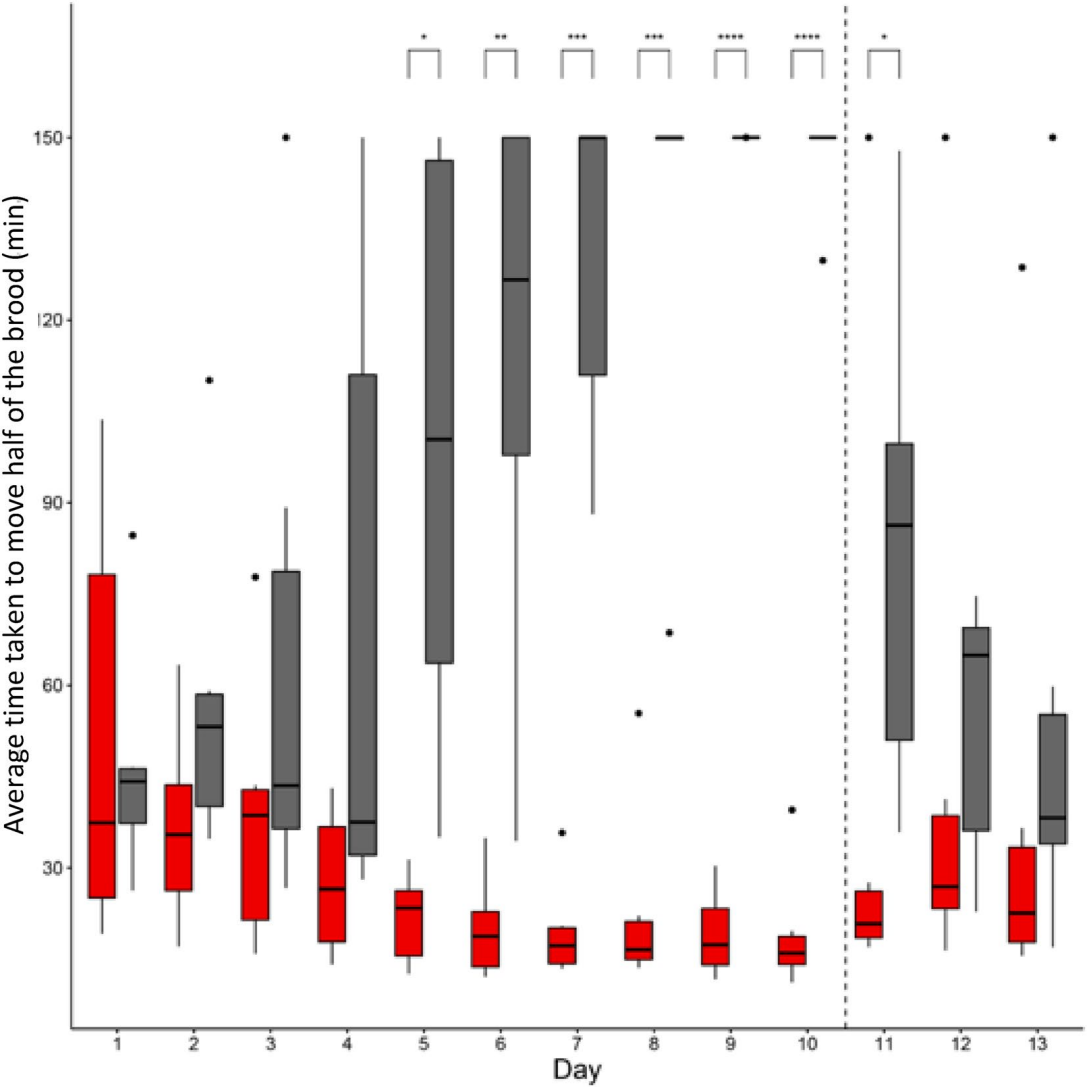
Pairwise comparisons for mean time taken to transport half the brood between control (red) and treatment (grey) subcolonies, per day. Significance is denoted by stars and additional statistical summary is available in Supplementary Table 5.

The time taken to transport half the brood significantly increased over the first ten days in treatment subcolonies (*t* = 6.263, *p* < 0.0001). By contrast, the time taken to transport half the brood significantly decreased over time in the control subcolonies (*t* = − 8.285, *p* < 0.0001) and from day five on, the time taken to move brood diverged significantly between control and treatment subcolonies (Fig. 9; *F*(9,45) = 13.931, *p* < 0.0001). Accordingly, over the 13 days of the experiment, the average time taken to transport brood was significantly lower in control than in treatment subcolonies (*F*(1,5) = 96.138,* p* < 0.0001).

**Figure 10:**
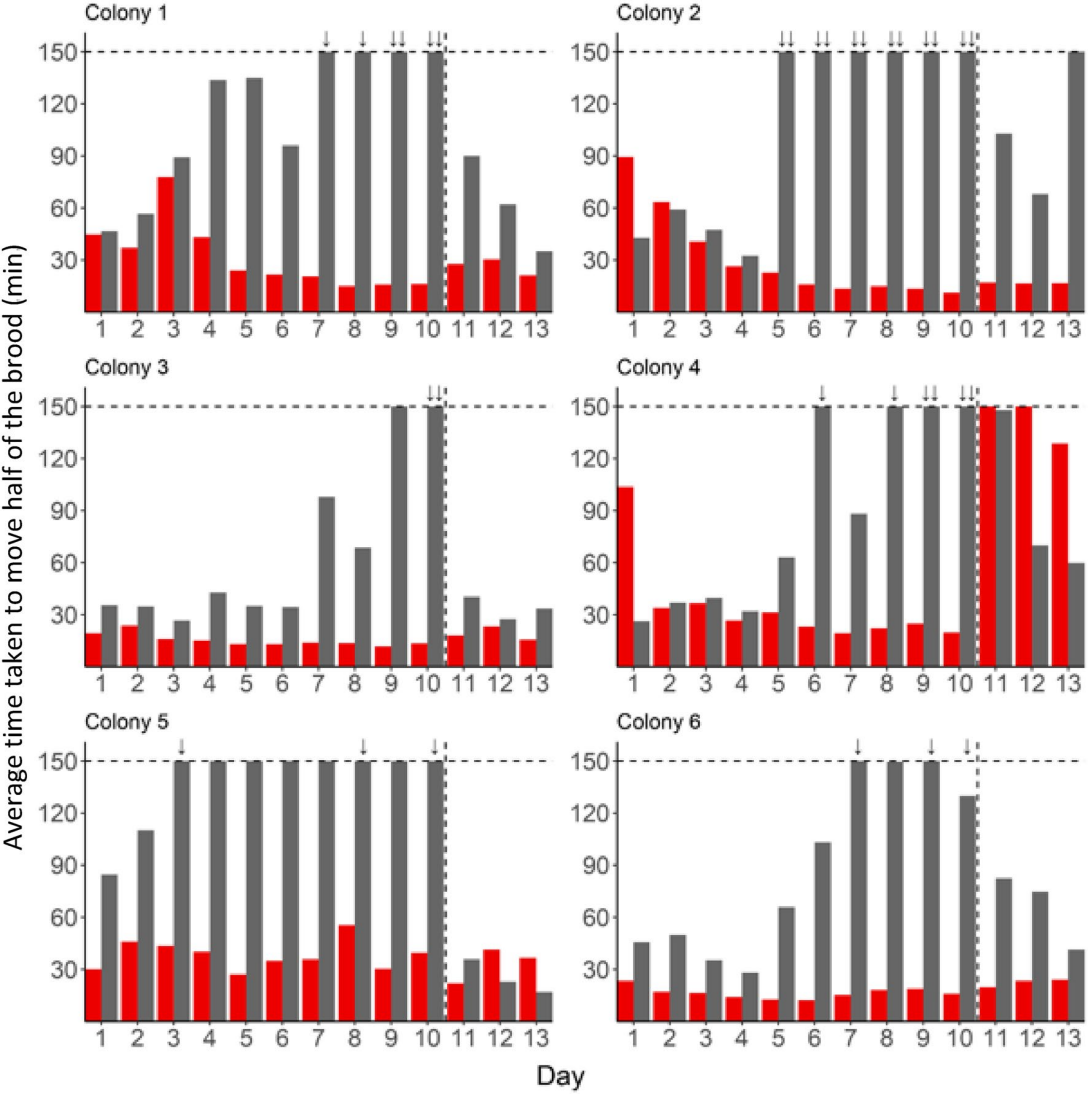
Mean time taken to transport half of the brood per day (average of the two observation sessions) for control (red) and treatment (grey) subcolonies. A time of 150 min was reported for observation sessions in which less than half of the brood was transported. Bars with a ↓ or ↓↓ indicate no brood transport occurred at all during either one or both observation sessions. Days 11, 12 and 13 present data following re-introduction of removed workers.

During each of the observation sessions of the first 10 days of the experiment brood transport always occurred in the control colonies. By contrast, in treatment subcolonies there were instances with no brood transport (Fig. 10) and over time there was a significant increase in the probability that brood would not be transported (*z* = − 4.97, *p* < 0.0001). Before brood transport stopped, the number of transporters that were removed varied between subcolonies with an average of 36.8% (range: 15–62%) of the workforce. Brood transport stopped as early as day 3, or as late as day 10, with an increase in the number of cases without brood transport over the time-period of the experiment. By day 10, none of the six treatment subcolonies transported brood during one or both observation sessions.

Following re-introduction of workers on day 11, brood transport started again in all treatment subcolonies (Fig. 10). The majority of workers (91.8%; range: 82.6–100%), which transported brood on day 11 in treatment subcolonies, were workers which had previously participated to brood transport during the first ten days. This was also observed on days 12 and 13 (average of the two days: 85.8%; range: 66.7–100%). This is consistent with data from control subcolonies where, once again, the majority of workers (88.2%; range 82.9–100%) transporting brood on day 11 were the same workers that had previously been transporting brood, although this value was a little lower on days 12 and 13 (72.2%; range 45.7–87.5%). Consequently, reintroduction of workers resulted in significantly faster transport of brood in treatment subcolonies when comparing days 8, 9, 10 to days 11, 12, 13 (*W* = 476.5, *p* < 0.005; Wilcoxon rank sum test), but did not affect the average time taken to transport brood in control subcolonies (*W* = 245, *p* = 0.15).

## Discussion

Our study reveals that only a small number of highly specialised workers performed most of the brood transport. This finding is consistent with observations on *Tetramorium erraticum*^59^ where a small number of specialist intranidal brood transporters were responsible for over a third of all brood transports. Similarly, during colony emigration in *Formica obscuripes*^60^ and *Formica exsecta*^61^ only a few workers were reported to consistently transport brood and in *Myrmica rubra* only 18–34% of the workers transported brood when nest emigrations were forced^62^. Even though there was strong specialisation for brood transport, we found no evidence that specialisation was associated with higher efficiency of brood transport. The speed of brood transport was not significantly correlated with either the frequency of brood transport or the total number of brood transported. Specialisation is usually assumed to enhance efficiency and has been well documented in ant species with highly polymorphic worker castes^10,20,21,63^. However, more recent reports on monomorphic species have demonstrated that task specialisation is not necessarily correlated with enhanced task efficiency^8,64,65^. Although evidence disputing the association between specialisation and efficiency such as ours is rare, we propose that future work should not automatically assume that specialised workers are any more efficient than their generalist counterparts, particularly amongst monomorphic species of the eusocial Hymenoptera.

A surprising finding of the study was that the removal of brood transporters ultimately stopped all brood transport across every treatment subcolony. This implied that the remaining workers were unable to flexibly re-allocate their tasks to transport brood. This is a particularly striking result, as ants are believed to constantly monitor the local climatic conditions of the nest and adjust the position of the brood to ensure optimal growth and development^3,66–68^. If maintaining optimal conditions for the brood is vital, flexibility of workers transporting brood should minimise risks to the brood, should specialist transporters be lost^25,69,70^. Indeed, behavioural flexibility is widespread across the eusocial Hymenoptera^71^ with multiple reports of flexible foraging^31,72,73^ and flexible nurse and brood care behaviour^22,27,33,35,74,75^. Nonetheless, there are examples where flexible task allocation is not found. In both *Pogonomyrmex badius*^36^ and *Camponotus fellah*^38^, the removal of foragers did not cause the remaining workers to flexibly re-allocate their tasks in compensation for the lack of foraging behaviour. Furthermore, limited behavioural flexibility may be advantageous when worker task re-allocation is unnecessary, such as when worker mortality is minimal and environmental perturbations are rare^76^. Since *C. floridanus* is restricted to the subtropical region of Florida (USA), an environment known for its stable and predictable temperature^76^, and because it is mostly nurses performing brood transport, which are unlikely to experience high mortality within the confines of their nest, this may explain why we found no behavioural flexibility in brood transport behaviour.

Our data also revealed that minor workers were more likely to transport brood than majors. As the primary role of major workers in *Camponotus* spp. is defensive^77–79^ with extremely limited brood care (e.g., *F. obscuripes*^60^), this is not surprising. When majors did transport brood, there was a negative correlation between size (headwidth and body length) and the likelihood to transport brood, such that small majors transported more brood compared to large majors. This suggests that smaller majors may have a more expanded behavioural repertoire than larger majors. However, neither headwidth nor body length correlated with the likelihood to perform brood transport in minor workers, which is consistent with what was observed in *A. sexdens*^47^. Furthermore, workers which were nurses were significantly more likely to transport brood than workers which were foragers. Our results support previous studies where workers performing brood transport have been identified as nurses^42,43,80,81^.

The vast majority of workers that transported brood to the new strip at 27 °C had first visited the strip and were therefore privately informed. On the occasions where workers transported brood before having visited the new strip at 27 °C, the time taken to transport their first brood was longer. This suggests that the workers were unaware of a suitable location to deposit the brood and had to search for one while transporting it. In addition, there was a strong positive correlation between the time that elapsed until workers were privately informed of the new strip at 27 °C and the time that elapsed until they transported their first brood, suggesting that this information acquisition was important in driving brood transport behaviour.

Our study revealed no evidence of social information (i.e., that privately informed workers were able to socially inform naïve workers and prompt brood transport to the new strip at 27 °C). There was no correlation between the time that elapsed until workers were socially informed of the new strip at 27 °C and the time that elapsed until they transported their first brood. This result supports the findings of Mersch et al.^46^, who also observed no communication between brood transporters in the ant *C. fellah*. Although several behaviours are performed without communication across ant species^82,83^, this finding is interesting because it is generally assumed that brood transport is a group response that involves some form of recruitment^45^.

In conclusion, this study provides new insights into the enigmatic process of brood transport^45^. We show that brood transport is a task performed by a few highly specialised minor workers found within the nurse community. We find no evidence that these specialists have enhanced efficiency and when removed, brood transport stops with no subsequent task re-allocation amongst the remaining workers. These findings suggest that the organisation of some ant colonies may be less sophisticated than previously believed, at least with regard to brood transport, but may ultimately have resulted in a stable behavioural system that requires only a few workers and little regulation to function.

### Supplementary Information

Supplementary Information 1.

Supplementary Information 2.

## Data Availability

All data generated or analysed during this study are included in this published article and its supplementary information files.
