# Supplementary material for: Targeted worker removal reveals a lack of flexibility in brood transport specialisation with no compensatory gain in efficiency
Source: Sci Rep. 2024 Feb 28;14:4850. doi: 10.1038/s41598-024-55244-w (PMC10901836; doi:10.1038/s41598-024-55244-w)
Supplement: Supplementary file 2 — Supplementary Information 2. [file 41598_2024_55244_MOESM2_ESM.pdf]

Supplementary Information

Targeted worker removal reveals a lack of flexibility in brood transport specialisation with no compensatory gain in efficiency

Sean McGregor, Fazil E. Uslu, Mahmut Selman Sakar, Laurent Keller

Supplementary Figures

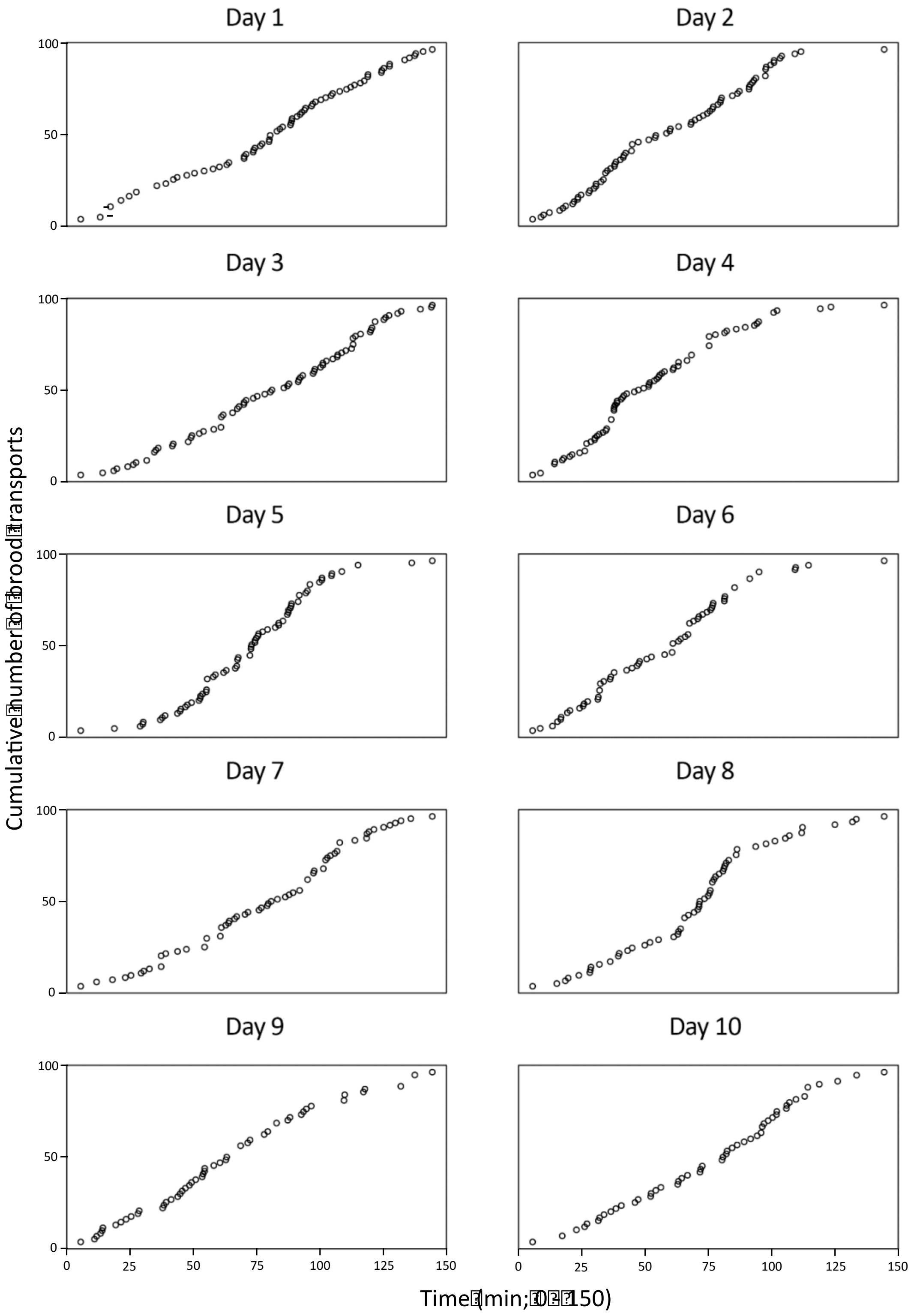

Supplementary Figure 1. *Individual brood transport events as a function of time for the first ten days during observation sessions from 07:00 to 09:30 UTC illustrating the sigmoid-esque function of the rate of brood transport.*

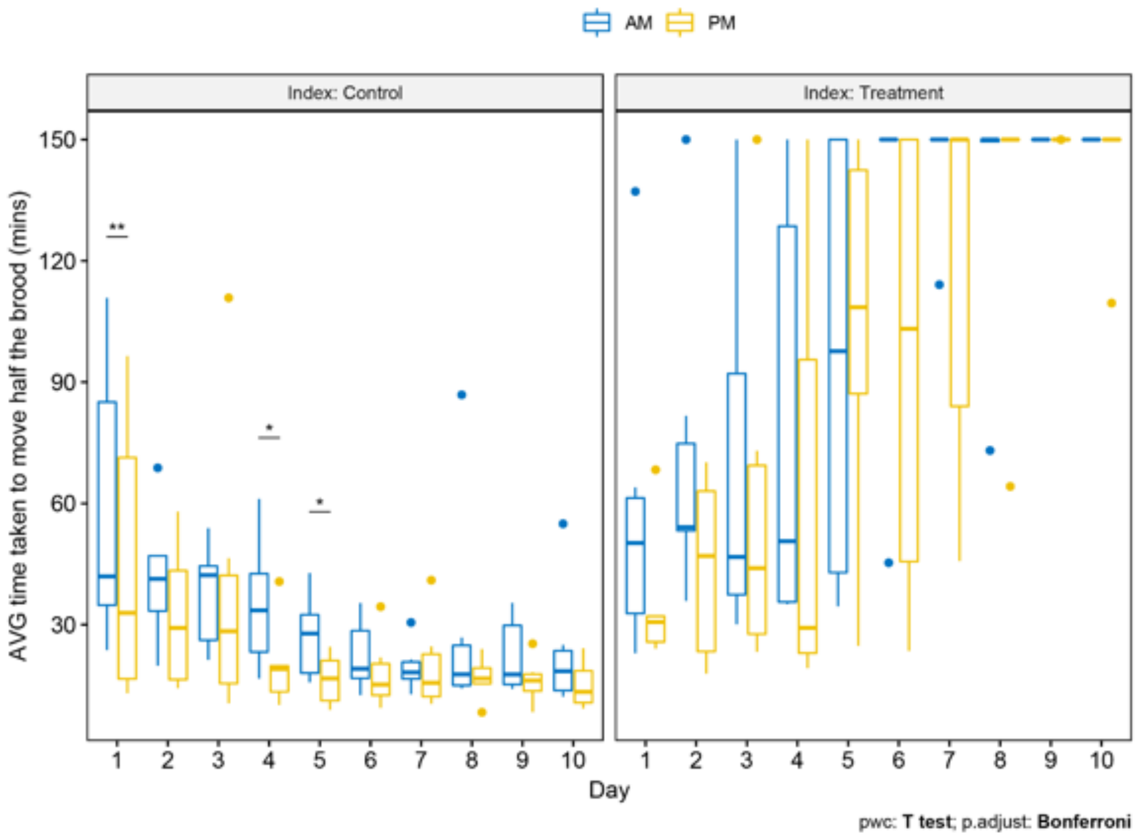

Supplementary Figure 2. *Average time taken to move half the brood for observation sessions initiated at 07:00 UTC (blue) or at 11:00 UTC (yellow), per day, for control and treatment subcolonies. Brood transport was faster during the second observation session (i.e., 11:00 UTC) than the first one (i.e., 07:00 UTC); only three pairwise comparisons are significant (day 1, 4 and 5 in the control). Additional statistical output supplied in Supplementary Table 4.*

Supplementary Tables

Supplementary Table 1. *Number of ants (n) transporting brood per control subcolony.*

Control Subcolony	n (>1 brood transport)	n (Infrequent)	n (frequent)	n (> half of all brood transports)
**1**	85	39	46	17
**2**	85	50	35	18
**3**	60	31	29	13
**4**	47	35	12	7
**5**	87	53	34	21
**6**	65	30	35	12

Supplementary Table 2. *Statistical regression between the number of observation sessions in which transporters moved brood and the average or total number of brood moved per observation session. The change in intercept (ΔIntr.) and slope (Δslope) is given when comparing the model using data for infrequent transporters with the model using data for frequent transporters.*

	Transporter type	Estimate	SE	t (Df)	p	ΔIntr.	Δslope
Average	Infrequent	0.29	0.08	3.59 (422)	<.0001	0.6	-0.16
	Frequent	0.13	0.03	4.06 (422)	<.0001		
Total	Infrequent	1.36	0.18	7.56 (422)	<.0001	3.04	-0.58
	Frequent	0.77	0.07	10.79 (422)	<.0001		

Supplementary Table 3. *Mean transport time per number of observation sessions or per total number of brood transported. We report both the statistical coefficients for infrequent and frequent transports, the change in intercept value (ΔIntr.) and the change in the regression estimate (Δslope).*

	Transporter type	Estimate	SE	t (Df)	p	ΔIntr.	Δslope
Number of obs. sessions	Infrequent	<0.01	<0.01	0.03 (422)	.99	-0.18	<-0.01
	Frequent	<0.01	0.02	0.02 (422)	.98		
Total number of brood transported	Infrequent	<-0.01	<0.01	-1.69 (422)	.09	-0.17	<0.01
	Frequent	<-0.01	<0.01	-1.02 (422)	.31		

Supplementary Table 4. *Pairwise comparisons for average time taken to move half the brood between control and treatment subcolonies per day per observation session. Results are consistent with the results presented in main text. Below, plot illustrating the significant pairwise comparisons.*

Day	Index	group1	group2	n_1	n_2	statistic	df	p.adj	
**1**	Control	AM	PM	6	6	5.018	5	.004	**
**2**	Control	AM	PM	6	6	2.309	5	.069	ns
**3**	Control	AM	PM	6	6	-0.129	5	.902	ns
**4**	Control	AM	PM	6	6	2.646	5	.046	*
**5**	Control	AM	PM	6	6	2.966	5	.031	*
**6**	Control	AM	PM	6	6	1.733	5	.144	ns
**7**	Control	AM	PM	6	6	-0.073	5	.945	ns
**8**	Control	AM	PM	6	6	1.249	5	.267	ns
**9**	Control	AM	PM	6	6	2.239	5	.075	ns
**10**	Control	AM	PM	6	6	1.634	5	.163	ns
**1**	Treatment	AM	PM	6	6	1.189	5	.288	ns
**2**	Treatment	AM	PM	6	6	1.738	5	.143	ns
**3**	Treatment	AM	PM	6	6	1.130	5	.31	ns
**4**	Treatment	AM	PM	6	6	2.508	5	.054	ns
**5**	Treatment	AM	PM	6	6	-0.606	5	.571	ns
**6**	Treatment	AM	PM	6	6	1.818	5	.129	ns
**7**	Treatment	AM	PM	6	6	1.464	5	.203	ns
**8**	Treatment	AM	PM	6	6	0.907	5	.406	ns
**9**	Treatment	AM	PM	6	6	<0.001	5	1	ns
**10**	Treatment	AM	PM	6	6	0.999	5	.364	ns

Supplementary Table 5. *Pairwise comparisons for average time taken to move half the brood between control and treatment subcolonies per day using the time taken to move half the brood as the independent variable.*

Day	group1	group2	n_1	n_2	statistic	df	p.adj	
**1**	Control	Treatment	6	6	0.196	5	.853	ns
**2**	Control	Treatment	6	6	0.030	5	.977	ns
**3**	Control	Treatment	6	6	-3.510	5	.017	*
**4**	Control	Treatment	6	6	-1.878	5	.119	ns
**5**	Control	Treatment	6	6	-4.777	5	.005	**
**6**	Control	Treatment	6	6	-5.677	5	.002	**
**7**	Control	Treatment	6	6	-9.308	5	.000241	***
**8**	Control	Treatment	6	6	-10.261	5	.000151	***
**9**	Control	Treatment	6	6	-10.568	5	.000131	***
**10**	Control	Treatment	6	6	-9.801	5	.000188	***

Supplementary Note

Construction of the ANT°C presented a number of engineering challenges. The following provides a detailed explanation on the design choices and development of the system as a modular addition to the pre-existing automated tracking system. Once an ANT°C was constructed installation is as simple as removing a section of the polystyrene base of a tracking box (Figure 1b), placing it over the aluminium strips and stabilising the system with the addition of support struts (Figure 1c).

Peltier elements

Peltier elements work as a heat pump utilising the principle of the Peltier effect. When an electron passes through an element it has to change its energetic level at each junction of the different metallic conductors and semiconductors, and consequently releases or absorbs heat (see illustration below).

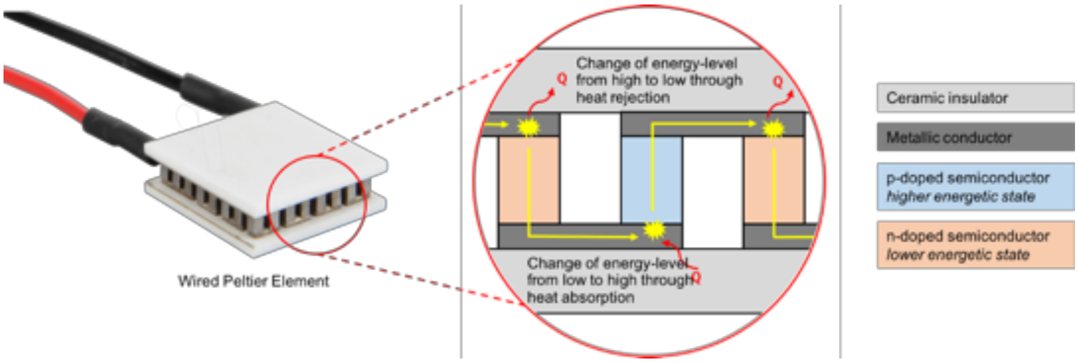

We chose to use Peltier elements to generate thermal patterns, as they are widely used in thermoelectric cooling and heating apparatus, are extremely stable, and permit rapid temperature change in a controlled system. However, their use requires accurate thermal calculation. The heat flux of a Peltier element ($Q^{\cdot}{}_{Peltier}$) can be expressed as:

$$Q^{\cdot}{}_{Peltier} = (\prod_A - \prod_B)\, I$$

Where $\prod_{A,B}$ are the Peltier coefficients of the conductors and I is the current passed through the element. The heat flux direction is invertible by inversing the current as it is proportional to I. This allows control for both heating and cooling. To find the equation for the overall heat flux from the cold side of the Peltier element to the hot side, we must calculate the effect of Joule heating (heat production through the Peltier element; $Q^{\cdot}{}_{Joule}$), which is a product of the current passed over the Peltier element and its resistance (R):

$$Q^{\cdot}{}_{Joule} = I^2 R$$

in addition to Fourier's law (the rate of heat transfer through a Peltier element; $Q^{\cdot}_{Fourier}$), which is a product of the Peltier elements thermal conductivity (k) and the temperature gradient between the cold and hot side (∇T):

$$Q^{\cdot}_{Fourier} = -k\nabla T$$

The overall equation is:

$$Q^{\cdot}_{cold\rightarrow hot} = Q^{\cdot}_{Peltier} + \frac{1}{2}Q^{\cdot}_{Joule} - \dot{Q}_{Fourier} = (\prod_A - \prod_B)\, I + \frac{1}{2}I^2R - k\frac{T_{hot} - T_{cold}}{e}$$

Where T_{hot} and T_{cold} are the temperatures of the hot and cold side of the Peltier elements and the $\frac{1}{2}$ factor in front of Joule heating represents symmetric dissipation of heat from both sides of the Peltier element. This heat dissipation, and the role of temperature gradient between hot and cold, are two important considerations when using Peltier elements. In order to construct a stable system, it demands 1) that we provide a heat sink on the side of the Peltier elements that is not the object of temperature control, and 2) that this heat sink maintains a stable temperature. To account for this, see Thermal control and heatsink design in the next section.

We utilised 5 proportional-integral-derivative (PID) thermos-electric (TEC) controllers (TEC-1122-SV; Meerstetter Engineering) to regulate current to Peltier elements. A printed circuit board connected below the ANT°C heatsink serially connects Peltier elements into ten groups of 6 with each controller independently generating a signal for control of two independent strips. Each strip had its own object sensor located at the midpoint of the 6 Peltier elements and an additional heat-sink temperature sensor for monitoring purposes so the user can be made aware of any thermal fluctuations with the heat-sink during usage, which could otherwise result in device failure. The object sensors used were PT100 (DM-31, Farnell) and combined with the TEC-Controllers provided accuracy of up to 0.01°C. All temperature changes were programmed using the TEC-service software (Meerstetter engineering, www.meerstetter.ch/customer-center/downloads). Prior to the use of the software, all Peltier elements were calibrated (see Peltier equation above) using both feedback from the PT100 object sensor, sink surface sensors, and an external heat sensor, which was itself calibrated on another aluminium surface of known temperature. A ramp of 0.5°C/s was specified for all temperature changes used in this project.

Thermal control and heatsink design

Ensuring the thermal stability of the ANT°C was an important design priority. However, it was also necessary to ensure independent thermal regulation of each of the ten strips. To that effect each of the aluminium strips was separated from one another with 3D printed insulating barriers (243 x 5 x

2mm) and each strip was affixed to the heatsink via insulating screws. These barriers prevented thermal transfer both between aluminium strips, and between aluminium strips and the heatsink, ensuring that only the Peltier elements themselves were controlling the temperatures defined by the user. In addition, Peltier elements were placed onto specially engraved 'slots' (Supplementary figure 5a) which partially encased the four edges of each Peltier element to allow optimal placement, but also prevented thermal 'leaching' between serially connected rows. The efficiency of these design choices was tested visually with a FLIR thermal camera (FLIR One) and confirmed using onboard PT100 sensors and a remote temperature sensor.

With a total of 60 Peltier elements per ANT°C, the amount of heat generated over time is substantial. Any deviation in the temperature of the heat-sink over time will ultimately result in failure of the device and so water cooling was a necessity of the design. However, as our control system of ten strips consists of 6 serially connected Peltier elements, it was not possible to provide uniform water cooling underneath all Peltier elements as water must flow either parallel or orthogonally (Supplementary Figure 3a and b, respectively) to the direction of strips. However, as we control each of the ten strips of Peltier elements independently an orthogonal water flow allows us to independently calibrate the performance of Peltier elements for each variation in T_{cold} underneath each series of 6 Peltier elements.

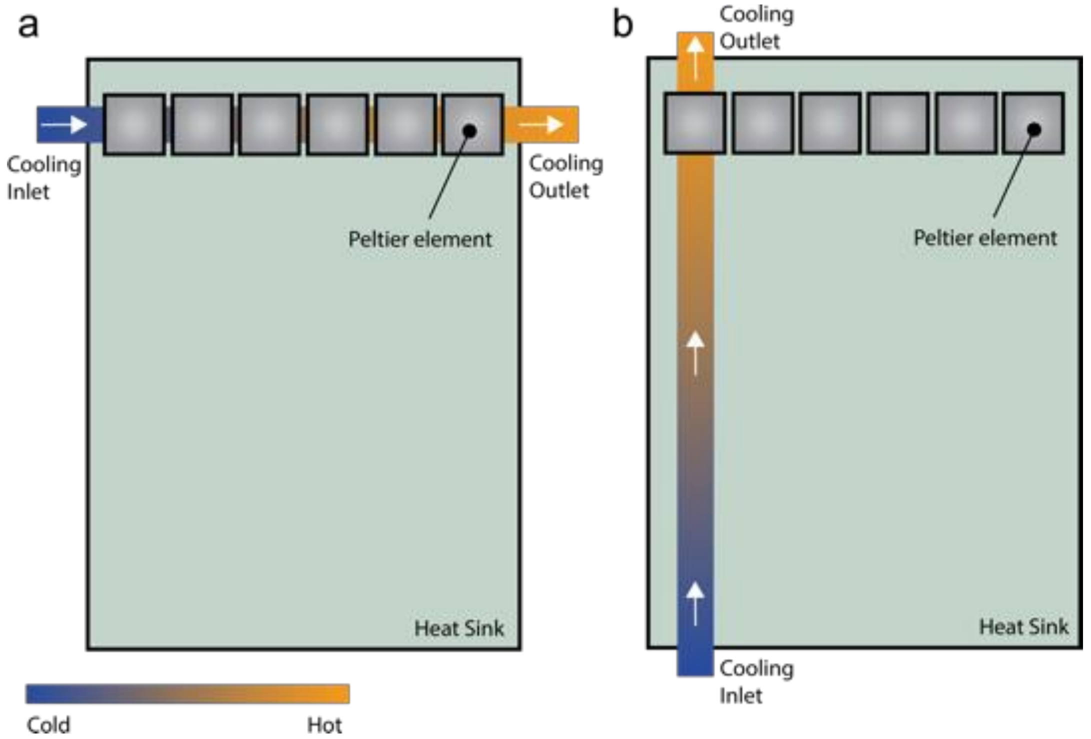

Supplementary Figure 3. *Cooling channel orientation was an important design consideration as neither (a) parallel or (b) orthogonal flow allows for uniform temperature distribution underneath the Peltier elements. However, whilst orthogonal flow means that T_{cold} varies for each independent strip of 6 Peltier elements, each Peltier within the series of 6 experiences the same value for T_{cold} allowing independent calibration for*

each strip. The identical functioning of each Peltier element within the strip provided homogenous water-cooling distribution.

In addition to orthogonal flow (Supplementary Figure 3b), it is necessary to calculate the flow rate of water throughout the heatsink to ensure uniformity. Failure to do so results in heterogenous flow velocity (e.g., Supplementary Figure 4a) which, when using an orthogonal flow scheme, will result in different observed T_{cold} values per Peltier element in a series. This would result in rapid failure of the device as the calculations for heat flux of multiple elements in a series would be incorrect, leading to heat accumulation over time, destabilising the system. To design a heat sink that would distribute water homogenously, we simulated a variety of designs (Supplementary Figure 4) in SimScale (SimScale GmbH; a cloud based finite element simulation software with the parameters and boundary conditions in Supplementary Table 6). After several iterations, we designed a system with two symmetrical lateral inlets with a ladder-like partitioning structure ending in three equally distanced outlets parallel to six cooling channels (Supplementary Figure 4d). This structure provides the most homogenous distribution of water throughout the heat sink.

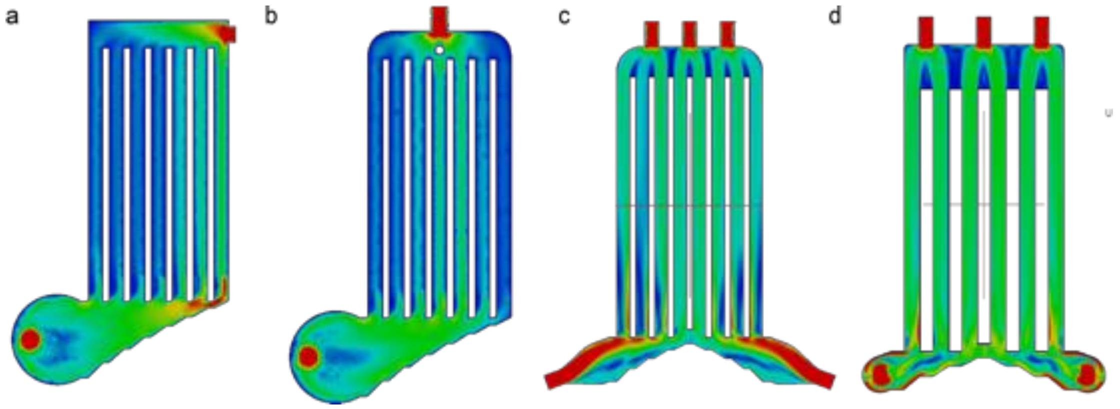

Supplementary Figure 4. *Iterations of cooling channel design with improving optimisation. Ultimately symmetric lateral inlets, multiple outlets and simple ladder-like design optimises water flow velocity and homogeneity of heat transfer. Colour grading according to simulated water velocity where red is fastest, and blue is slowest. Simulations generated using SimScale (SimScale Gmbh).*

Supplementary Table 6. Parameters and conditions specified for SimScale simulations over the course of iterative design improvements.

**Material**	Water
**Viscosity model**	Newtonian
**Turbulence model**	k-omega shear stress transport (SST)
**Steady-state or Transient**	Steady-state (after 1000s)
**Wall boundary condition**	No slip
**Inlet boundary condition**	0.0004m^3/s
**Outlet boundary condition**	0.0004m^3/s
**Post-processing**	Cut through idle (velocity scale: 0 – 0.8m/s)

The heatsink was then manufactured by milling an aluminium block and consists of two layers, a thicker upper layer (400 x 276 x 30mm) that contains the grooves for the placement of all 60 Peltier elements and passageways for connecting cables on one side and engraved cooling channels on the other (Supplementary Figure 5). Cooling channels were rectangular as this provides a greater surface area contact with the underside of the Peltier elements and are easier to mill. The lower layer (400 x 276 x10mm) was then affixed over the side with the cooling channels to complete construction. To prevent any possible water leakage in the sealing process, O-rings were placed at every cable passageway between the two layers of the heatsink (Supplementary Figure 6). The heat-sink was then connected via PVC tubing to a refrigerated water cooler (Model 157-5251 Arctic circulator, ThermoFisher) set to 12°C.

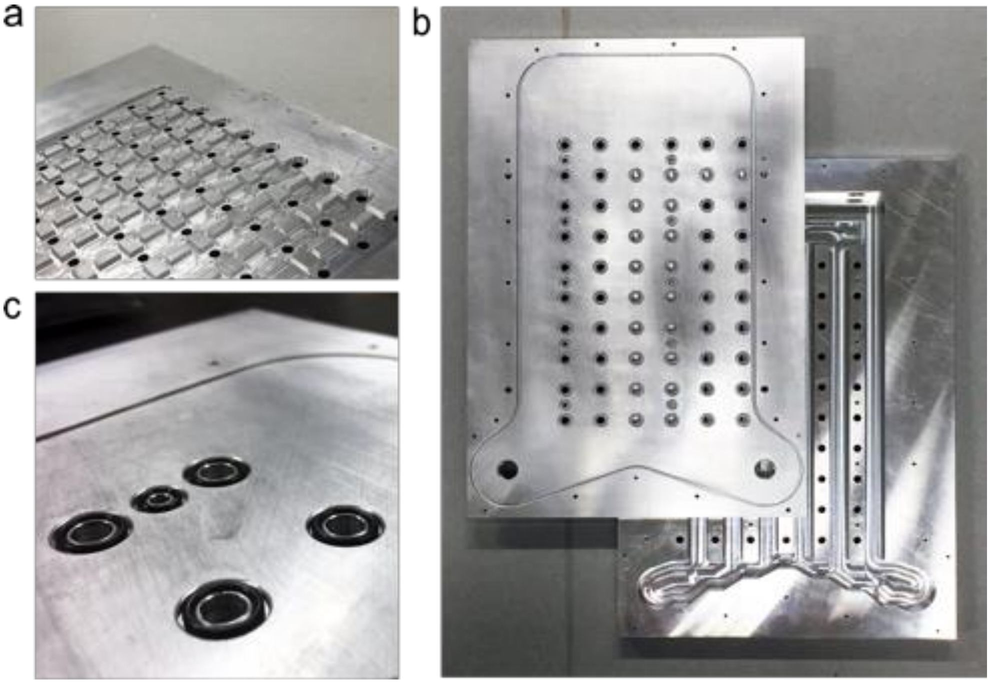

Supplementary Figure 5. *Final design of the heatsink. a) Grooves for ensuring optimal placement of, and to thermally isolate serially connected Peltier elements. Cable passageways are also visible. b) Lower layer of heat sink positioned above upper layer with optimised engraved cooling channels visible. c) Sealing O-rings placed within cable passageways to protect against potential water leakage during sealing.*

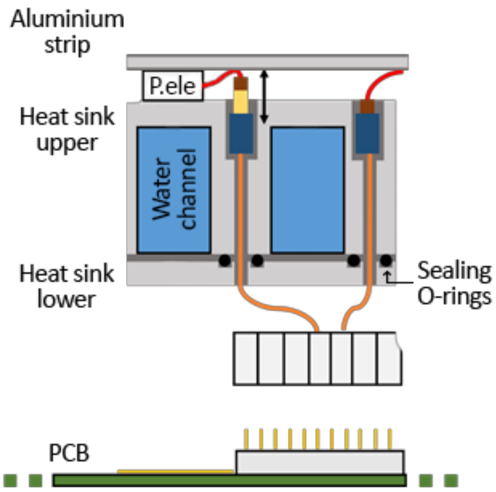

Supplementary Figure 6. *A cross-sectional schematic of the ANT°C. Cabling of the Peltier elements (P.ele) are clipped and passed through to adjustable connectors for ease of replacement in case of malfunction during initial calibration. Cable passageways pass through the upper and lower layers of the heat sink to a pin connector mounted on an integrated PCB. Sealing O-rings are placed at the join between upper and lower layers of the heat-sink to ensure no leakage when the two layers are sealed. Water channels are rectangular to improve heat exchange from the straight, flat Peltier element to the water.*
